# Effect of Zr Content on Phase Stability, Deformation Behavior, and Young’s Modulus in Ti–Nb–Zr Alloys

**DOI:** 10.3390/ma13020476

**Published:** 2020-01-19

**Authors:** Kyong Min Kim, Hee Young Kim, Shuichi Miyazaki

**Affiliations:** 1 Graduate School of Pure and Applied Sciences, University of Tsukuba, Tsukuba, Ibaraki 305-8573, Japan; antnom@gmail.com; 2 Faculty of Pure and Applied Sciences, University of Tsukuba, Tsukuba, Ibaraki 305-8573, Japan; 3 Foundation for Advancement of International Science, Tsukuba, Ibaraki 305-0821, Japan; 4 Center of Advanced Innovation Technologies-VŠB-Technical University of Ostrava, 17. listopadu 15, 708 00 Ostrava-Poruba, Czech Republic

**Keywords:** Ti alloys, Young’s modulus, martensitic transformation, shape memory effect, superelasticity

## Abstract

Ti alloys have attracted continuing research attention as promising biomaterials due to their superior corrosion resistance and biocompatibility and excellent mechanical properties. Metastable β-type Ti alloys also provide several unique properties such as low Young’s modulus, shape memory effect, and superelasticity. Such unique properties are predominantly attributed to the phase stability and reversible martensitic transformation. In this study, the effects of the Nb and Zr contents on phase constitution, transformation temperature, deformation behavior, and Young’s modulus were investigated. Ti–Nb and Ti–Nb–Zr alloys over a wide composition range, i.e., Ti–(18–40)Nb, Ti–(15–40)Nb–4Zr, Ti–(16–40)Nb–8Zr, Ti–(15–40)Nb–12Zr, Ti–(12–17)Nb–18Zr, were fabricated and their properties were characterized. The phase boundary between the β phase and the α′′ martensite phase was clarified. The lower limit content of Nb to suppress the martensitic transformation and to obtain a single β phase at room temperature decreased with increasing Zr content. The Ti–25Nb, Ti–22Nb–4Zr, Ti–19Nb–8Zr, Ti–17Nb–12Zr and Ti–14Nb–18Zr alloys exhibit the lowest Young’s modulus among Ti–Nb–Zr alloys with Zr content of 0, 4, 8, 12, and 18 at.%, respectively. Particularly, the Ti–14Nb–18Zr alloy exhibits a very low Young’s modulus less than 40 GPa. Correlation among alloy composition, phase stability, and Young’s modulus was discussed.

## 1. Introduction

Metallic materials such as Ti alloys, cobalt-chromium based alloys and stainless steels have been extensively used as structural biomaterial. One of the critical issues associated with metallic biomaterials is their high Young’s modulus because the large difference of the elastic modulus between metallic implants and adjacent bone tissues can lead to stress shielding, causing bone resorption and osteoporosis [1,2]. Among metallic biomaterials, Ti and its alloys have explicitly received more attention due to not only their balanced combination of excellent mechanical properties and biocompatibility, but also relatively lower Young’s modulus than stainless steels and cobalt-chromium based alloys [3,4,5]. However, when compared with the Young’s modulus of bone tissues (10–30 GPa), commercially pure Ti (CP-Ti) and Ti–6Al–4V, which are the most commonly used Ti based alloys for biomedical applications, possess a considerably higher Young’s modulus of about 110 GPa.

Over the last decades, there have been extensive studies to reduce Young’s modulus of Ti alloys as much closer to those of bone tissues [6,7,8,9,10,11,12,13,14,15,16,17]. Ti alloys have two stable phases, α phase with a hexagonal close-packed (hcp) crystal structure and β phase with a body centered cubic (bcc) structure and they are classified into three main categories according to major constituent phases: α-type, (α + β)-type, and β-type Ti alloys. Among them, β-type Ti alloys have been confirmed to have the lowest Young’s modulus, and thus they have attracted increasing research attention in recent years. Up to date, many β-type Ti alloys have been developed, such as Gum metal [18], Ti–Nb–Ta–Zr [7,11,19,20], Ti–Nb–Sn [21,22,23,24], Ti–Nb–Zr [9,25,26,27,28,29,30], Ti–Nb–Hf [31] and Ti–Nb–Zr–Sn [32,33]. It is noted that most β-type Ti alloys exhibiting low Young’s modulus contain Nb as a β stabilizing alloying element due to the moderate β phase stabilizing ability and biocompatibility.

It has been confirmed that the Young’s modulus of β-type Ti alloys is strongly dependent on the stability of the β phase [6,9,20,33,34,35,36,37,38]. Generally, Young’s modulus decreases as the β phase becomes unstable. However, the decrease in the stability of the β phase stimulates the formation of α′′ martensite phase and ω phase, leading to the increase in Young’s modulus. In order to assess the stability of the β phase and to optimize the alloy composition, various approaches have been proposed, including *Mo* equivalent (*Mo_eq_*), electron to atom ratio (*e*/*a*), and *d*-electron alloy design theory. The *Mo* equivalent is an empirical parameter representing the contribution of alloying elements on the stability of β phase in comparison to that of Mo. Although *Mo_eq_* has been widely used as a guideline to design β-type Ti alloys, there have been some controversial issues concerning the effect of alloying elements on the stability of β phase and modification has been continued [39]. The average number of valence electrons per atom or electron to atom ratio (*e*/*a*) is also a representative measure of the elastic constants of bcc crystals. It has been reported that as a decrease in the value of *e/a*, the shear modulus *c′* = (*c*_11_ − *c*_12_)/2 and bulk modulus *B* of a bcc crystal decrease, causing the β phase to become unstable [6,9,20,33,34,35,36,37,38]. The *d*-electron alloy design theory is based on molecular orbital calculations. Two key parameters of this theory are the bond order (*Bo*) and the d-orbital energy level (*Md*) which are calculated for each alloying element. *Bo* is parameter to show the overlapping of the electron clouds of adjacent two atoms, which is a measure of the covalent bond strength between Ti and alloying element. *Md* is found to be closely related with electronegativity and atomic radius of each alloying element. The average values of *Bo* and *Md*, calculated by taking the compositional averages, have been utilized to predict phase boundaries and the stability of the β phase. Experimental results have validated that the β phase becomes unstable with decreasing *Bo* or with increasing *Md* [40,41,42,43]. However, it has been pointed out that the phase boundary line in the *Bo-Md* map shifts as the change of constituent alloying elements [42,44].

β-type Ti alloys have also attracted attention as biomedical shape memory alloys [38,45,46,47,48,49,50,51,52]. The phase stability of the β phase is a key factor governing shape memory effect and superelasticity in β-type Ti alloys because they are related to martensitic transformation from the β phase to the orthorhombic α′′ martensite phase. It has been also reported that the *Bo-Md* map for Ti alloys is useful to predict the martensitic transformation temperature and deformation mechanism [53,54,55,56,57]. Despite such extensive research, much uncertainty still exists on the relations among Young’s modulus, martensitic transformation behavior, and the values of *e*/*a*, *Bo*, and *Md*, and the experimental data are still insufficient to understand the mechanisms involved. This study focuses on the effect of the Zr addition on the phase stability and Young’s modulus in Ti–Nb alloys because Zr has been used as a major alloying element in β-type Ti alloys for biomedical applications due to its superior biocompatibility [2,9,11,25,26,27,28,29,30,32,33]. It has been demonstrated that Zr suppresses the martensitic transformation from the β phase to the α′′ phase and enhances the stability of the β phase of Ti–Nb alloys [32,38,58]. Furthermore, the addition of Zr in Ti–Nb alloys can modify the values of *Bo* and *Md* without changing *e*/*a*. Ti–Nb–Zr alloys with various Nb and Zr contents were fabricated and the composition dependence of phase constitution and deformation behavior was investigated. The phase boundary between the β phase and the α′′ martensite phase in Ti–Nb–Zr alloys was clarified. The relations among phase stability, martensitic transformation behavior, and Young’s modulus were analyzed. Finally, a novel guideline to design β-type Ti alloys with low Young’s modulus was proposed.

## 2. Materials and Methods

**Figure 1:**
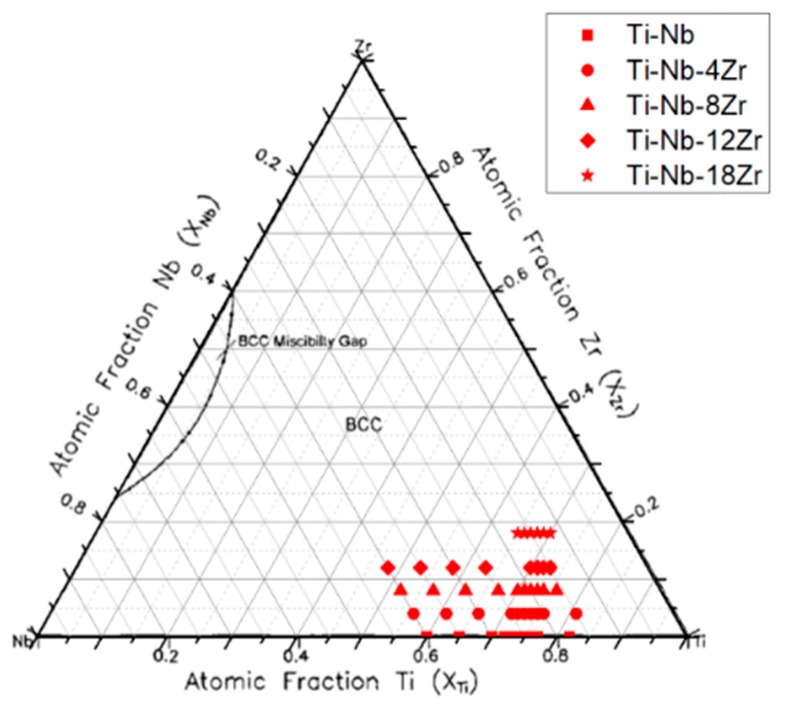
Alloy compositions investigated in this study in the isothermal sections of the Ti–Nb–Zr phase diagram at 1173 K [59].

A total of 44 alloys were investigated in this study. The alloys are named in their Nb content and Zr content. For binary Ti–Nb alloys, ten compositions with different Nb content from 18 to 40 at.%, which are denoted as Ti–(18–40)Nb, were investigated. For Ti–Nb–Zr ternary alloys, 4 different series of alloys containing 4 at.% Zr, 8 at.% Zr, 12 at.% Zr or 18 at.% Zr were investigated where the range of Nb content is indicated in parenthesis. All the alloy compositions investigated in this study are indicated in the isothermal sections of the Ti–Nb–Zr phase diagram at 1173 K (Figure 1). Ti–(18–40)Nb, Ti–(15–40)Nb–4Zr, Ti–(16–40)Nb–8Zr, Ti–(15–40)Nb–12Zr, Ti–(12–17)Nb–18Zr alloys were fabricated by the arc melting method. Unless specified otherwise, values of alloy content are in atomic percent (at.%) hereafter. The alloy ingots were melted on a water-cooled Cu hearth in an argon atmosphere in the form of small button weighing about 20 grams which was approximately 25 mm in diameter and 10 mm in height. The ingots were sealed in a quartz tube under vacuum and were subjected to a homogenization treatment at 1273 K for 7.2 ks. Then, the ingots were cold rolled into plates of approximately 0.5 mm in thickness with a final reduction ratio of 95%. Specimens for X-ray diffraction (XRD), microstructure analysis, and tensile tests were cut from the cold rolled sheets by an electrical discharge machine. These specimens were annealed at 1173 K for 0.3 ks in Ar-filled quartz tubes and quenched in ice water by breaking the quartz tubes. After annealing, the surface contamination of specimens was cleaned using methanol and acidic solution.

XRD analysis was performed using a Rigaku Smartlab instrument (Tokyo, Japan) with Cu Kα radiation (40 kV, 30 mA). Microstructural characterization was performed using a scanning electron microscope (JSM-IT300; JEOL, Tokyo, Japan). Tensile tests were carried out along the rolling direction using dog-bone specimens with a 20-mm gauge length and 1.5-mm width at a strain rate of 0.005 mm/s at room temperature. The strain of the specimens was measured using a non-contacting video extensometer (TRViewX; Shimadzu, Kyoto, Japan) with two targets. Transformation temperatures were evaluated from differential scanning calorimetry (DSC) curves. The DSC measurements were performed at heating rate of 10 K/min in the Shimadzu DSC-60 (Kyoto, Japan).

## 3. Results and Discussion

### 3.1. Phase Constitutions

**Figure 2:**
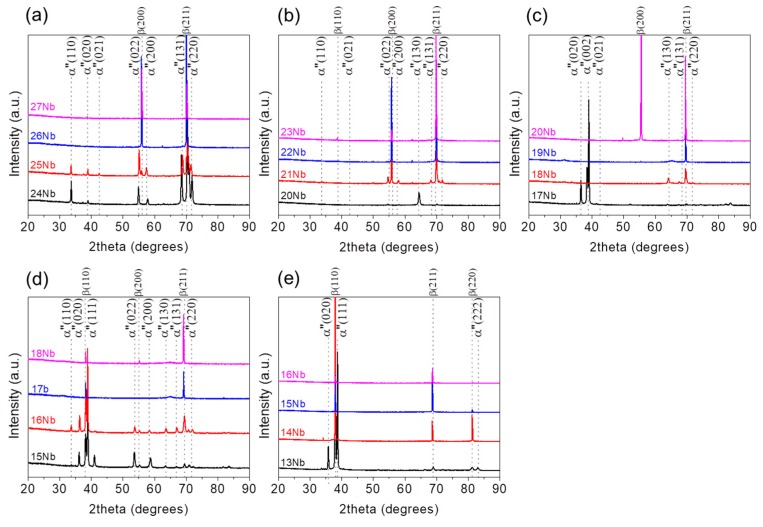
XRD profiles of Ti–Nb–Zr alloys obtained at room temperature: (**a**) Ti–(24–27)Nb; (**b**) Ti–(20–23)Nb–4Zr; (**c**) Ti–(17–20)Nb–8Zr; (**d**) Ti–(15–18)Nb–12Zr; (**e**) Ti–(13–16)Nb–18Zr alloys.

**Figure 3:**
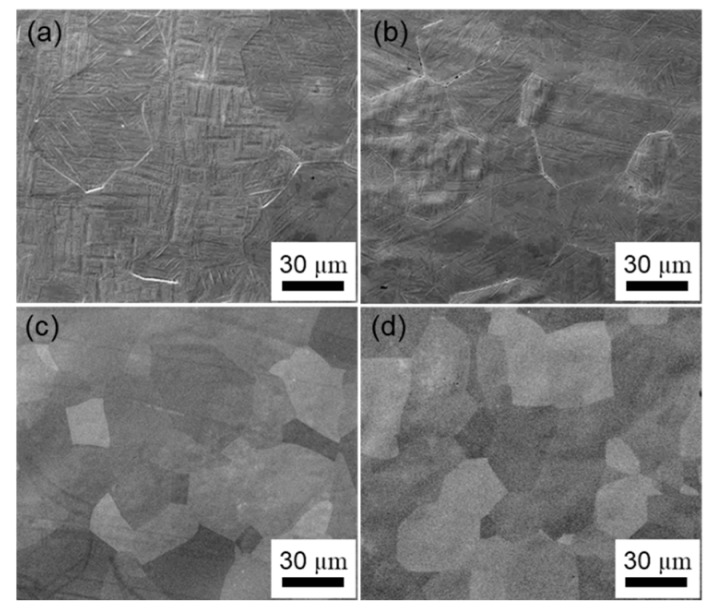
SEM micrographs of Ti–Nb–12Zr alloys: (**a**) Ti–15Nb–12Zr; (**b**) Ti–16Nb–12Zr; (**c**) Ti–17Nb–12Zr; (**d**) Ti–18Nb–12Zr alloys.

Phase constitutions of Ti–Nb–Zr alloys annealed at 1173 K for 0.3 ks were investigated by XRD at room temperature. Figure 2 shows XRD profiles of selected alloys to identify the critical concentration of Nb to obtain a single β phase for binary Ti–Nb and ternary Ti–Nb–(4, 8, 12, 18) Zr alloys. For the binary Ti–Nb alloys (Figure 2a), the peaks from both α′′ martensite phase with an orthorhombic structure and β phase were observed in the alloys with lower Nb content, such as Ti–24Nb and Ti–25Nb alloys. On the other hand, Ti–26Nb and Ti–27Nb alloys exhibited a single β phase, implying that the martensitic transformation start temperature (*M*_s_) decreases with increasing Nb content and becomes below room temperature when the Nb content is 26 at.%. In consequence, it is clear that the lower limit content of Nb to suppress the martensitic transformation and to obtain a single β phase is 26 at.% in the Ti–Nb binary alloys, which is consistent with previous reports [45,60]. For the Ti–Nb–4Zr alloys (Figure 2b), peaks from both the α” martensite phase and the β phase were detected in Ti–20Nb–4Zr and Ti–21Nb–4Zr alloys while there are only peaks from the β phase in XRD profiles of Ti–22Nb–4Zr and Ti–23Nb–4Zr alloys, indicating that the lower limit content of Nb to obtain a single β phase decreased to 22 at.% by the addition of 4 at.% Zr. As seen in Figure 2, as the increased in Zr content from 4 at.% to 8 at.% to 12 at.% and to 18 at.%, the critical Nb content to reduce the *M*_s_ below room temperature, i.e., to obtain a single β phase at room temperature, further decreased from 22 at.% to 19 at.%, 17 at.% and 14 at.%, respectively. Figure 3 shows scanning electron microscopy (SEM) micrographs of Ti–(15–18)Nb–12Zr alloys as representative examples. The SEM micrographs are well consistent with XRD results shown in Figure 2d. Martensite plates are clearly seen in Ti–15Nb–12Zr and Ti–16Nb–12Zr alloys, on the other hand, a single-phase structure of the β phase is seen in Ti–17Nb–12Zr and Ti–18Nb–12Zr alloys. These results are consistent with the previous reports that Zr decreases *M*_s_ and plays a role to stabilize the β phase [15,48,59]. Within the range of alloy compositions investigated, it is noted that Zr has an impact on the decrease in *M*_s_ of Ti–Nb alloys, which is equivalent to about two thirds of Nb.

### 3.2. Mechanical Properties

**Figure 4:**
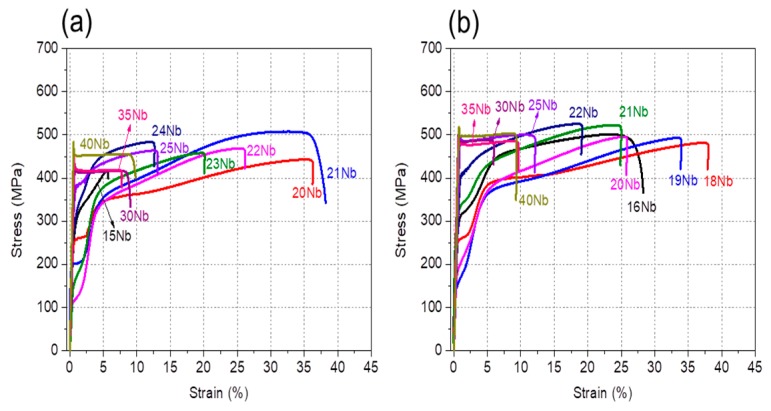
Stress-strain curves of Ti–Nb–Zr alloys obtained at room temperature: (**a**) Ti–Nb–4Zr alloys; (**b**) Ti–Nb–8Zr alloys.

**Figure 5:**
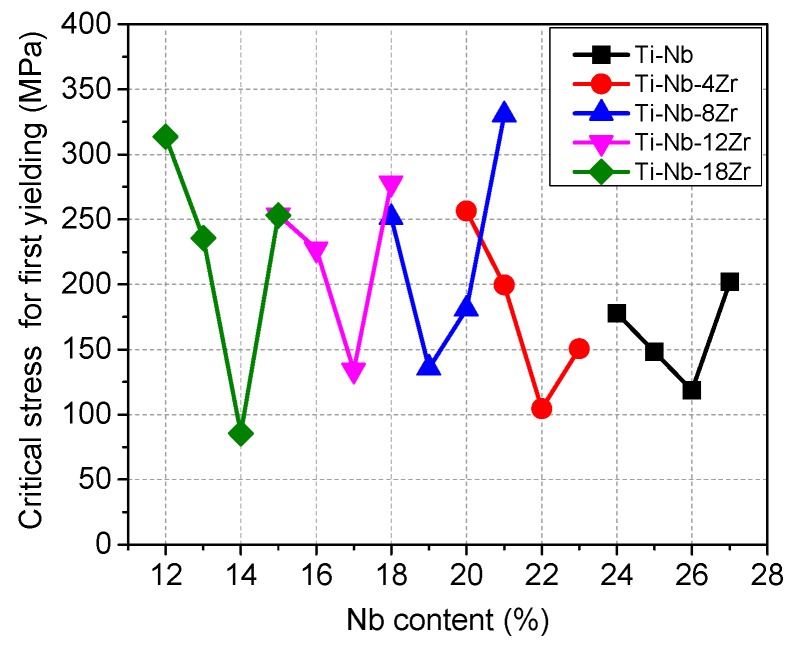
Nb content dependence of the critical stress for the first yielding for Ti–Nb and Ti–Nb–Zr alloys with various Zr content.

Mechanical properties and deformation behavior are also strongly dependent on Nb and Zr contents. Figure 4 shows examples of tensile stress-strain curves for (a) Ti–Nb–4Zr and (b) Ti–Nb–8Zr alloys obtained at room temperature. The yield strength, ultimate tensile strength and elongation of all alloys are listed in Table S1. For Ti–Nb–4Zr alloys, the alloys with low Nb content (Ti–(15–25)Nb–4Zr) exhibited double yielding while the alloys with more than 25 at.% Nb revealed single yielding. As shown in Figure 2b, the Ti–Nb–4Zr alloys containing 22 at.% Nb and more consisted of a single β phase, therefore the first yielding in the Ti–(22–25)Nb–4Zr alloys is due to the stress induced martensitic transformation. On the other hand, the first yielding in the Ti–(15–21)Nb–4Zr alloys is believed to be due to the reorientation of α” martensite variants. It is noted that the critical stress for the first yielding decreases with increasing Nb content, reaching a minimum of 110 MPa at 22 at.% Nb and then increases again with further increasing Nb content. It is also noted that elongation shows a decreasing tendency with increasing Nb content. A large elongation in the alloys with low Nb content is supposed to be due to transformation-induced plasticity (TRIP) and twinning-induced plasticity (TWIP) effects [53,54,55,56,61]. As shown in Figure 4b, stress-strain curves of Ti–Nb–8Zr alloys exhibits a similar dependence on Nb content as that shown for the Ti–Nb–8Zr alloys. Figure 5 shows the Nb content dependence of the critical stress for the first yielding for the Ti–Nb–Zr alloys with various Zr content. It is also noted that the yield stress takes a minimum value at the compositions locate near the phase boundary of (β + α′′)/β, which is reasonable to consider that the stress for inducing martensitic transformation decreases with decreasing stability of the β phase and takes a minimum value at the phase boundary.

**Figure 6:**
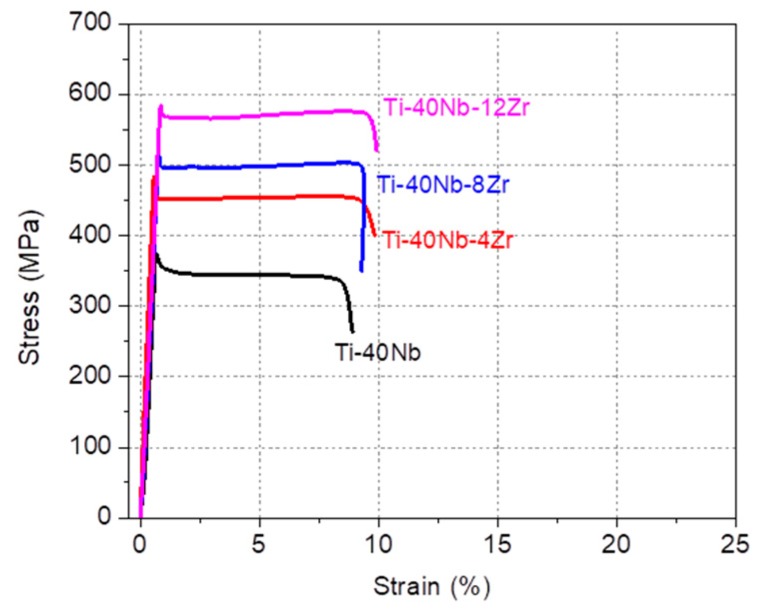
Stress-strain curves of Ti–40Nb and Ti–40Nb–(4, 8, 12) Zr alloys obtained at room temperature.

The stress–strain curves of Ti–40Nb and Ti–40Nb–(4, 8, 12) Zr alloys which have fully stabilized β phase are compared in Figure 6 to assess the strengthening effect of Zr. The yield strength increased almost linearly with increasing Zr content: from 375 MPa for the Ti–40Nb alloy to 525 MPa for the Ti–40Nb–8Zr alloy, and to 590 MPa for the Ti–40Nb–12Zr alloy, respectively. Accordingly, it is evident that, although the effect is not very strong, Zr has a strengthening effect in Ti-Nb alloys.

### 3.3. Deformation Behavior and Martensitic Transformation Temperature

**Figure 7:**
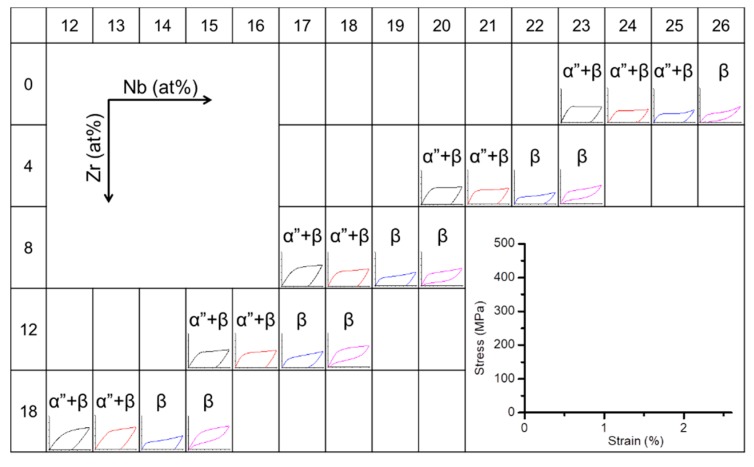
Stress-strain curves of Ti–40Nb and Ti–40Nb–(4, 8, 12) Zr alloys obtained at room temperature.

Figure 7 shows stress-strain curves obtained during a loading-unloading cycle for Ti–Nb and Ti–Nb–Zr alloys with the compositions near phase boundary of (β + α′′)/β. After unloading the specimens were heated to investigate reverse transformation and shape recovery. In Ti–Nb alloys, shape memory effect was observed in the Ti–(23–25)Nb alloys; most of strain was recovered by heating the unloaded specimen. The Ti–26Nb alloy exhibited partial superelasticity and partial shape memory effect. Clear superelasticity was observed in the Ti–23Nb–4Zr, Ti–20Nb–8Zr, Ti–18Nb–12Zr, Ti–15Nb–18Zr alloys. The decrease in the Nb content exhibiting superelasticity with the increase in Zr content is reasonable considering that Zr acts as the β phase stabilizing element in Ti–Nb alloys and decreases the martensitic transformation temperature. The results of Zr and Nb content dependences of shape memory properties are consistent with previous reports [48,51,59].

**Figure 8:**
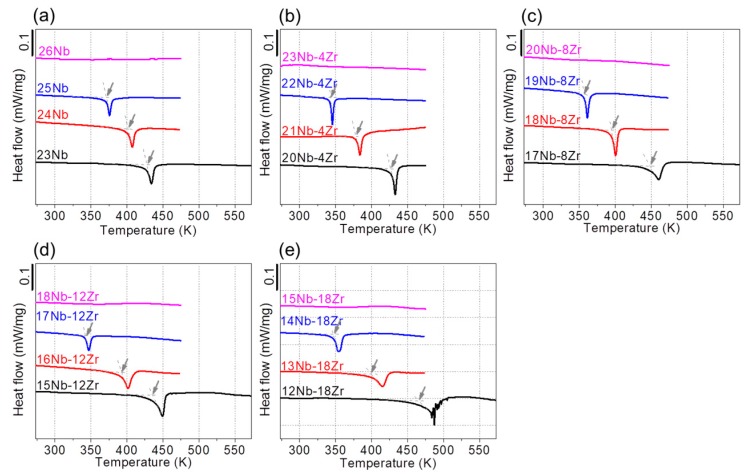
DSC curves of the Ti–Nb and Ti–Nb–Zr alloys upon heating: (**a**) Ti–(23–26)Nb; (**b**) Ti–(20–23)Nb–4Zr; (**c**) Ti–(17–20)Nb–8Zr; (**d**) Ti–(15–18)Nb–12Zr; (**e**) Ti–(12–15)Nb–18Zr alloys.

**Figure 9:**
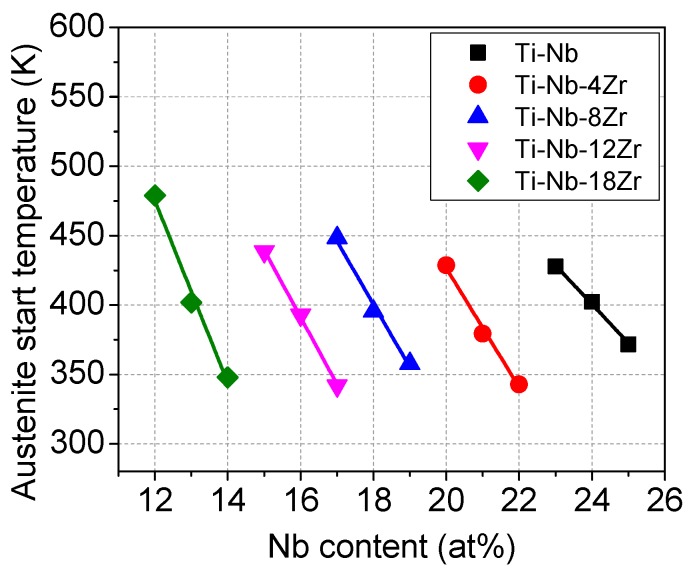
Nb content dependence of the *As* temperature for Ti–Nb and Ti–Nb–Zr alloys.

In order to clarify the effect of Nb and Zr content on the reverse transformation temperature, DSC measurements were performed by heating samples taken from the specimens that had been loading–unloading tested, and the results are shown in Figure 8. No peak was detected in the alloys that showed superelasticity upon loading–unloading tests, i.e., Ti–26Nb, Ti–23Nb–4Zr, Ti–20Nb–8Zr, Ti–18Nb–12Zr, and Ti–15Nb–18Zr. This is reasonable by considering that superelasticity occurs at temperature higher than the reverse transformation temperature and thus the reverse transformation occurs upon unloading at room temperature. On the other hand, all the alloys that showed shape memory effect exhibited a distinct endothermic peak upon heating, which is associated with the reverse transformation from the α′′ phase to the β phase. The reverse transformation start temperature (*A*_s_) for the Ti–Nb–Zr alloys with various Zr content is plotted as a function of the Nb content in Figure 9. It is seen that the *A*_s_ temperature decreases with increasing Nb content with a slope of −28 K/1 at.% Nb for the binary Ti–Nb alloys. The slope became steeper with increasing Zr content, namely from −43 K/1 at.% Nb for the Ti–Nb–4Zr alloys to −66 K/1 at.% Nb for the Ti–Nb–18Zr alloys. It is also noted that the Ti–24Nb, Ti–18Nb–8Zr, Ti–16Nb–12Zr, and Ti–13Nb–18Zr alloys have almost similar values of 400 K, indicating that the impact of Zr on decreasing *A*_s_ temperature is equivalent to about two thirds times that of Nb. This result is in good agreement with microstructural observation and the composition dependence of deformation behavior.

### 3.4. Young’s Modulus

**Figure 10:**
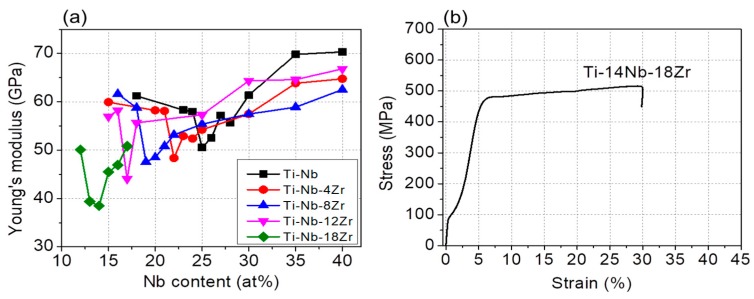
(**a**) Nb content dependence of the Young’s modulus for Ti–Nb and Ti–Nb–Zr alloys, and (**b**) stress-strain curve of Ti–14Nb–18Zr.

Young’s moduli of the Ti–Nb and Ti–Nb–Zr alloys were evaluated using the stress-strain curves and they are plotted as a function of Nb content in Figure 10a. For the Ti–Nb alloys, Young’s modulus gradually decreased with decreasing Nb content, reaching a minimum Young’s modulus of 51 GPa at 25Nb and then increased again with further decreasing Nb content. The Ti–Nb–Zr alloys exhibited similar trends of the Nb content dependence on Young’s modulus; but the Nb content taking the minimum value of Young’s modulus was shifted to lower values as the increase in the Zr content. It is noted that Young’s moduli of Ti–Nb–Zr alloys with Zr contents 4, 8, 12, and 18 at.% take minimum values at Nb contents of 22, 19, 17, and 14 at.%, respectively, which locate near the phase boundary of (β + α′′)/β in a way similar to the dependence of the critical stress for the first yielding on the Nb content as shown in Figure 5. It is also worth noting that, when compared the minimum Young’s modulus of the series of alloys with different Zr content, Young’s modulus decreased with increasing Zr content to a very low value of 39 GPa for the Ti–14Nb–18Zr alloy. As shown in Figure 10b, the Ti–14Nb–18Zr alloy exhibited the stress induced martensitic transformation at a low stress level less than 100 MPa which is due to the low stability of the β phase.

**Figure 11:**
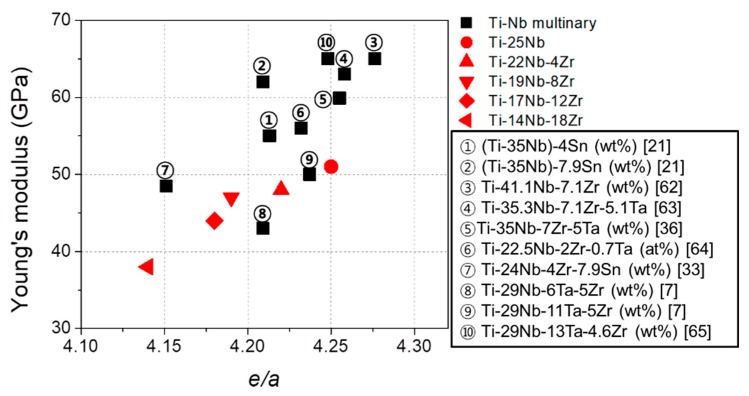
Comparison of Young’s modulus of the Ti–Nb based alloys as a function of *e*/*a*.

Previous studies have demonstrated that *e/a* is a dominant factor governing the elastic constants and Young’s modulus of bcc transition metals including β-type Ti alloys [20,33,35]. In order to understand the effect of Zr content on the lowest Young’s modulus for each series of alloys with different Zr content, the Young’s moduli of the Ti–25Nb, Ti–22Nb–4Zr, Ti–19Nb–8Zr, Ti–17Nb–12Zr and Ti–14Nb–18Zr alloys are plotted in Figure 11 as a function of *e*/*a*. For comparison purposes, the results of some β-type Ti alloys [7,21,33,36,62,63,64,65] developed for low Young’s modulus alloys are included in Figure 11. Note that *e*/*a* of the alloys investigated in this study decreased with increasing Zr content as follows: Ti–25Nb, 4.25; Ti–22Nb–4Zr, 4.22; Ti–19Nb–8Zr, 4.19; Ti–17Nb–12Zr, 4.17; and Ti–14Nb–18Zr, 4.14. The decrease is attributed to the fact that Zr decreases *M*_s_ of the alloys, and the addition of Zr shifts the phase boundary of (β + α′′)/β toward lower Nb content. Although the data were somewhat scattered, there is a clear tendency of decreasing Young’s modulus with decreasing *e*/*a* in accordance with the previous reports [20,33,35,66]. As a result, it is suggested that Zr is an effective alloying element in reducing Young’s modulus because it decreases the lower limit of *e*/*a* to maintain the β phase. Similarly, Sn is considered as a useful alloying element because it also decreases the *M*_s_ of the alloys while keeping *e*/*a*. These results may have important implications for developing useful guidelines for alloy design.

It has been demonstrated that Young’s modulus of the β phase is governed by elastic constants of *c′* and *c*_44_ [35]. Very recently, Kwasniak et al. [67] reported that the elastic constants of *c′* and *c*_44_ and lower limit of Young’s of Ti–Nb based alloys are dependent on electronic hybridization of electronic structures. They also proposed that the addition of a second transition metallic element can be useful for reducing Young’s modulus by tuning atomic bonding structure and elastic constants. It is suggested that Zr is a promising alloying element to control atomic bonding structure without increasing *e*/*a*. Further studies are required to evaluate the impact of Zr on the electronic structure of multicomponent β-Ti alloys and its effect on the elastic constants and Young’s modulus.

### 3.5. Phase Boundary of Ti–Nb–Zr Alloys in the Bo-Md Map

**Figure 12:**
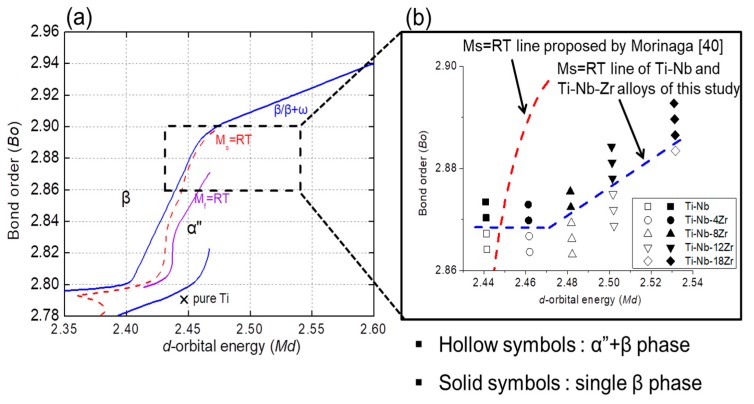
(**a**) *Bo-Md* map taken from Reference [41], and (**b**) newly constructed *Ms* line in this study.

As was mentioned in the introduction, *Bo*-*Md* maps have been used successfully as guideline for alloy design of Ti alloys. Figure 12a shows a *Bo*-*Md* map representing phase boundaries, where *M*_s_ = RT line corresponds to the phase boundary of (β + α′′)/β. The alloys investigated in this study, i.e., Ti–(24–27)Nb, Ti–(20–23)Nb–4Zr, Ti–(16–20)Nb–8Zr, Ti–(14–19)Nb–12Zr, and Ti–(13–16)Nb–18Zr alloys, are represented in Figure 12b, where the alloys consisting of only single β phase are indicated by solid symbols and the alloys consisting of both α′′ and β phases are denoted by hollow symbols. It is seen that the phase boundary of (β + α′′)/β for the Ti–Nb–Zr alloys is displaced toward a higher *Md* region from the line suggested by Morinaga et al. [40] and Abdel-Hady et al. [41]. These results are in agreement with previous studies which showed that Zr shifts the phase boundary downward in the *Bo-Md* map [9,41,42]. It is also worth mentioning that Young’s modulus of the alloys located on the phase boundary of (β + α′′)/β decreased as the *Md* increased.

## 4. Conclusions

In this study, the effects of the Nb and Zr contents on phase constitution, transformation temperature, deformation behavior, and Young’s modulus in Ti–(12–40)Nb–(0–18)Zr alloys were investigated. The main conclusions are as follows:

(1) The addition of Zr decreases the martensitic and reverse transformation temperatures of Ti–Nb alloys. The influence of Zr on decreasing the transformation temperatures is weaker, i.e., about two thirds that of Nb. The minimum Nb content to maintain the β phase at room temperature continuously decreases with increasing Zr content.
(2) Mechanical properties and deformation behavior strongly depend on Nb and Zr contents. Deformation behavior changes from double yielding to single yielding with increasing Nb or Zr content. The critical stress for the first yielding takes a minimum value at the composition locating near the phase boundary of (β + α′′)/β. For Ti–Nb–Zr alloys with fully stabilized β phase, the yield stress increased with increasing Zr content.
(3) Young’s modulus gradually decreases with decreasing Nb content, reaching a minimum value, and then increases again with further decreasing Nb content. The Nb content taking the minimum value of Young’s modulus was shifted to lower values as the increase in the Zr content. The Ti–25Nb, Ti–22Nb–4Zr, Ti–19Nb–8Zr, Ti–17Nb–12Zr and Ti–14Nb–18Zr alloys exhibit the lowest Young’s moduli among Ti–Nb–Zr alloys with Zr contents of 0, 4, 8, 12, and 18 at.%, respectively. The minimum Young’s modulus decreases with increasing Zr content. Particularly, the Ti–14Nb–18Zr alloy exhibits a very low value of 39 GPa.
(4) The addition of Zr in Ti–Nb alloys reduces the lower limit of *e*/*a* to maintain the β phase. The addition of Zr shifts the phase boundary of (β + α′′)/β downward in the *Bo-Md* map. Young’s modulus of Ti–Nb–Zr alloys located on the phase boundary of (β + α′′)/β decreases as the *Md* increases.

## Supplementary Materials

The following are available online at https://www.mdpi.com/1996-1944/13/2/476/s1, Table S1: Yield strength (YS), ultimate tensile strength (UTS) and elongation (EL) of Ti-Nb-Zr alloys.
