# Supplementary material for: Effect of Zr Content on Phase Stability, Deformation Behavior, and Young’s Modulus in Ti–Nb–Zr Alloys
Source: Materials (Basel). 2020 Jan 19;13(2):476. doi: 10.3390/ma13020476 (PMC7014103; doi:10.3390/ma13020476)
Supplement: Supplementary file 1 [file materials-13-00476-s001.pdf]

materials

SupplementaryMaterials

Effect of Zr Content on Phase Stability, Deformation Behavior, and Young's Modulus in Ti–Nb–Zr Alloys

Kyong Min Kim [1], Hee Young Kim [1,2,*] and Shuichi Miyazaki [2,3,4,*]

1 Graduate School of Pure and Applied Sciences, University of Tsukuba, Tsukuba, Ibaraki 305-8573, Japan; antnom@gmail.com
2 Faculty of Pure and Applied Sciences, University of Tsukuba, Tsukuba, Ibaraki 305-8573, Japan
3 Foundation for Advancement of International Science, Tsukuba, Ibaraki 305-0821, Japan
4 Center of Advanced Innovation Technologies-VŠB-Technical University of Ostrava, 17. listopadu 15, 708 00 Ostrava-Poruba, Czech Republic
* Correspondence: heeykim@ims.tsukuba.ac.jp (H.Y.K.); miyazaki@ims.tsukuba.ac.jp (S.M.)

Table S1. Yield strength (YS), ultimate tensile strength (UTS) and elongation (EL) of Ti-Nb-Zr alloys.

Ti–Nb				Ti–Nb–4Zr				Ti–Nb–8Zr				Ti–Nb–12Zr				Ti–Nb–18Zr			
**Nb (%)**	**YS (MPa)**	**UTS (MPa)**	**EL (%)**	**Nb (%)**	**YS (MPa)**	**UTS (MPa)**	**EL (%)**	**Nb (%)**	**YS (MPa)**	**UTS (MPa)**	**EL (%)**	**Nb (%)**	**YS (MPa)**	**UTS (MPa)**	**EL (%)**	**Nb (%)**	**YS (MPa)**	**UTS (MPa)**	**EL (%)**
18	303	429	37	15	284	345	5	16	321	532	21	15	254	459	20	12	314	598	12
23	226	467	33	20	58	441	34	18	251	486	37	16	227	477	21	13	236	571	16
24	178	438	26	21	58	508	36	19	136	491	37	17	134	532	41	14	86	521	29
25	148	412	22	22	48	470	27	20	181	497	18	18	278	519	33	15	253	527	39
26	119	420	13	23	53	456	21	21	330	519	26	25	514	526	7	16	422	562	30
27	202	456	14	24	308	485	13	22	404	528	18	30	529	543	8	17	491	591	17
28	301	455	12	25	372	457	12	25	490	502	12	35	556	572	8				
30	369	394	8	30	426	426	9	30	481	495	4	40	590	576	8				
35	375	375	7	35	438	439	7	35	478	497	8								
40	375	377	5	40	478	479	11	40	525	524	12								
